# Why Harmless Sensations Might Hurt in Individuals with Chronic Pain: About Heightened Prediction and Perception of Pain in the Mind

**DOI:** 10.3389/fpsyg.2016.01638

**Published:** 2016-10-25

**Authors:** Tanja Hechler, Dominik Endres, Anna Thorwart

**Affiliations:** ^1^Department of Clinical Psychology for Children and Adolescents, University of TrierTrier, Germany; ^2^Department of Psychology, Philipp University of MarburgMarburg, Germany

**Keywords:** interoception, interoceptive predictive coding, chronic pain, pain perception, Bayes theorem, active inference

## Abstract

In individuals with chronic pain harmless bodily sensations can elicit anticipatory fear of pain resulting in maladaptive responses such as taking pain medication. Here, we aim to broaden the perspective taking into account recent evidence that suggests that interoceptive perception is largely a construction of beliefs, which are based on past experience and that are kept in check by the actual state of the body. Taking a Bayesian perspective, we propose that individuals with chronic pain display a heightened prediction of pain [prior probability *p(pain)*], which results in heightened pain perception [posterior probability *p(pain|sensation)*] due to an assumed link between pain and a harmless bodily sensation [*p(sensation|pain)*]. This pain perception emerges because their mind infers pain as the most likely cause for the sensation. When confronted with a mismatch between predicted pain and a (harmless bodily) sensation, individuals with chronic pain try to minimize the mismatch most likely by active inference of pain or alternatively by an attentional shift away from the sensation. The *active inference* results in activities that produce a stronger sensation that will match with the prediction, allowing subsequent perceptual inference of pain. Here, we depict heightened pain perception in individuals with chronic pain by reformulating and extending the assumptions of the interoceptive predictive coding model from a Bayesian perspective. The review concludes with a research agenda and clinical considerations.

## Case Example

Sarah, a 13 year-old girl has been suffering from visceral pain for over a year after she suffered from severe acute abdominal inflammation. In addition, she has developed a profound fear and anxiety of pain, especially in situations in which pain occurred in the past (e.g., in school). Furthermore, whenever she becomes aware of visceral sensations, she immediately interrupts her activities. She may then lie down or take pain medication^1^.

The girl in the case example suffers from chronic abdominal pain accompanied by fear and anticipatory anxiety of pain. Importantly, she adopts protective responses in situations that might not actually be painful, e.g., already when becoming aware of interoceptive (visceral) sensations. Interoception is defined as sensing changes in physiological sensations from inside the body including among others pain, temperature, itch, muscular and visceral sensations (Cameron, 2001; Craig, 2002; Tsay et al., 2015).

One important question is why Sarah adopts these protective but often maladaptive responses. One answer could be that Sara learned to expect pain in similar situations and her behavior is part of an anticipatory response (Vlaeyen and Linton, 2012). We like to discuss a different explanation. From a Bayesian viewpoint, it is possible and perhaps even inevitable that Sarah displays a heightened perception of pain when confronted with harmless interoceptive sensations. She then reacts to the perceived pain. Our hypothesis is in line with the interoceptive predictive coding model (Seth et al., 2011; Seth, 2013; Barrett and Simmons, 2015) and linked to the assumption of heightened interoceptive predictions in anxiety-prone individuals (Paulus and Stein, 2006, 2010).

## Interoceptive Predictive Coding

While intuition suggests that sensations cause perception, recent evidence suggests that the brain predicts sensory input, so as to make inferences about the causes of the sensations (Dayan et al., 1995; Rao and Ballard, 1999; Friston and Kiebel, 2009; Paulus and Stein, 2010; Barrett and Simmons, 2015). What we perceive therefore depends heavily upon the predictions of the brain, which reflect what the system already knows about the world and about the body. These predictions not only precede sensations, they determine sensation (Hawkins and Blakeslee, 2004). Brains are thus conceived as prediction machines that function according to the Bayesian interpretation of probability that balance prior expectations against new sensory evidence (Clark, 2013). The Bayesian perspective makes it furthermore possible to develop computational simulations of predictive coding strategies to reproduce and explain observed effects. Take the example of Buchel et al. (2014), who estimate the level of perceived pain of participants, taking into account their prior knowledge and experiences in the context of placebo analgesia. In this paper, we apply the Bayes theorem to account for pain perception in situations where non-painful sensory input emerges.

In Bayesian terms, pain perception is quantified as the posterior probability of pain given the sensations, *p(pain|sensations)*:

(1) $$\begin{array}{c} p(pain|sensations)=p(sensations|pain)*p(pain)/p(sensations) \end{array}$$

where *p(sensations)* is the prior probability of the sensations. The posterior *p(pain|sensations)* on the left hand side, and thus the perception of pain, increases with the the pain prediction [*p(pain)*], which may be conditional on past events.

The posterior depends also on the likelihood *p(sensations|pain)*. This likelihood might be heightened via (longer lasting) learning processes or by “active inference” within a situation. The former involves learning that the probability of perceiving a sensation is high given pain, even for harmless sensations that are not caused by pain. The latter describes a process with the objective of actively generating sensations with an already high likelihood *p(sensations|pain)*.

Both active inference and learning follow from the free energy principle, which posits that brains try to minimize sensory prediction errors. This can be achieved by either learning correct predictions, or by correcting mismatched sensory states by changing sensory input through action (Friston, 2009; Edwards et al., 2012). We will suggest that pain may be perceived in situations where non-painful sensory input emerges, in part, due to a pernicious failure of sensory attenuation such that individuals actively solicit or attend to sensory cues that are consistent with their predictions that they are in pain.

Interoception can be seen as resulting from this probabilistic, knowledge-driven inference on the causes of sensory signals. Interoceptive sensations are combined with prior probabilities (predictions) of causes, estimated from past experience, to create posterior probabilities that quantify beliefs about the causes of such interoceptive sensations in the present. This process is called interoceptive predictive coding (Seth, 2013; Barrett and Simmons, 2015). Specifically, it is assumed that there is an interoceptive system in the brain in which agranular visceromotor cortices generate visceromotor as well as viscerosensory predictions (Barrett and Simmons, 2015). These sensory predictions, which are themselves based on prior experiences and perceptions, function as hypotheses about the state of the body that can be tested against sensory signals that arrive in the brain.

If the pain prediction sufficiently anticipates the sensory input, the *perceptual inference* can be made that the hypotheses about the current state of the body are correct, i.e. the posterior *p(pain|sensations)* increases. In case of a prediction error, i.e., a discrepancy between the predicted and the sensory input, the prediction error signals may be relayed back to the agranular visceromotor cortices, where they serve to change the hypotheses about the state of the body to fit the sensory input, i.e., decrease the posterior *p(pain|sensations)* and by this the next prior *p(pain)*. This process is therefore also a *perceptual inference*. Alternatively, the brain can initiate sensory states that are in line with the prediction, in the sense that the sensory input fits with the prediction (Seth, 2013; Farb et al., 2015). This process is the already mentioned *active inference*. Third, the brain’s cognitive control networks can change the focus of attention by biasing the influence of incoming sensory input (attentional shift; Barrett and Simmons, 2015), e.g., by reducing its precision (Edwards et al., 2012).

## Why Individuals With Chronic Pain Perceive Pain When Harmless Sensory Input Emerges: Heightened Prediction and Perception of Pain in the Mind of Individuals With Chronic Pain

There is ample evidence that pain can be amplified through expectations of intense pain and reduced through expectations of pain relief (Tracey, 2010). This influence of expectations is usually assumed to be rooted in altered sensory processing and expectancy-related modulations (Eippert et al., 2009). The contribution of predictive coding and the specific role of perceptual and active inferences in pain perception and more specifically in chronic pain patients are to our knowledge less discussed. As an exception, Wiech et al. (2014) investigated the influence of altered perceptual decision-making (inference) compared to the influence of altered sensory processing in a probabilistic cueing paradigm. Individuals were confronted with cues signaling varying probabilities for the application of a high intensity versus low intensity stimulus. Results revealed that this prior information biased perceptual-decision making. This is one of the few studies which confirm that prior information can change pain perception by impacting on perceptual decision-making. In the following, we will elaborate on our assumption that individuals with chronic pain perceive pain even when confronted with harmless bodily sensations because the mind infers pain as the most likely cause for the sensation.

**FIGURE 1:**
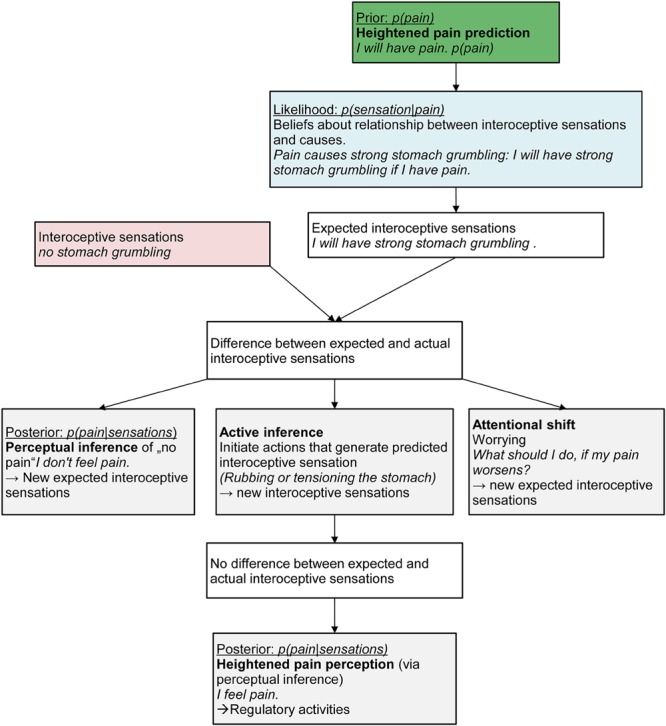
**The path from heightened prediction to perception of pain in the mind of Sarah, a 13-year old girl suffering from chronic abdominal pain**.

We posit that in chronic pain patients, pain prediction [*p(pain)*] is higher than in normal persons. Predictions or expectancies have been discussed as a core feature of mental disorders such as anxiety disorders (Paulus and Stein, 2006; Grupe and Nitschke, 2013; Rief et al., 2015). The assumption is that individuals with panic disorder display a heightened prediction of aversive outcomes (‘If my heart beats, I will die.’) which results in an exaggerated anticipatory response to interoceptive stimuli - even those that are not predictive of aversive states (Paulus and Stein, 2006; Farb et al., 2015). Similarly, we assume that individuals with chronic pain display a heightened and inaccurate pain prediction in situations where interoceptive sensations emerge that have been previously associated to pain. This will result in a conditioned fear response even when the sensation is not painful (De Peuter et al., 2011; Vlaeyen, 2015). Thus, a heightened prediction is not a novel assumption *per se*. However, we broaden the perspective by incorporating it in a Bayesian account of pain perception and posit that it will additionally lead to a heightened and inaccurate pain perception in the mind of individuals with chronic pain when faced with harmless sensory input (**Figure 1**).

Take again the example of Sarah. One day, she might have an exam at school and walks to the bus stop. She sees the bus arriving and runs quickly. She perceives mild stitches and breathlessness (sensory input). We assume that Sarah will display a heightened pain prediction [*p(pain)*]. These heightened pain predictions may have formed via associative learning processes (interoceptive fear conditioning; Bouton et al., 2001; Vlaeyen and Linton, 2012; Zaman et al., 2015), via biological processes such as structural brain changes due to ongoing pain experiences (Erpelding et al., 2014), via exposure to early infant pain experiences such as burn injuries or neonatal nociceptive input resulting in exaggerated expectations of pain in later life (Victoria and Murphy, 2015), or via social influences, e.g., anxious parental reactions to the child’s pain. Once Sarah has inferred and perceived pain, this heightened pain predictions might also emerge as a result of the perception itself as the posterior always becomes the prior for the next perceptual inference.

We also assume that Sarah has learned that pain leads to certain interoceptive sensations [likelihood *p(sensations|pain)*]. In the present situation, Sarah’s mind will therefore predict incoming interoceptive sensations in line with this expected pain, for example strong stomach grumbling, because she has learned that pain is associated with strong stomach grumbling. This will result in divergent observed body state (no stomach grumbling) and predicted body state (strong stomach grumbling). Sarah could now infer that the predicted pain is actually not happening [low posterior *p(pain|sensations)*] and generate an alternative causal explanation for the sensory input (breathlessness), e.g., “physical strain is the cause for the breathlessness.” This would also lead to a diminished pain prediction in the next instance [low prior *p(pain)*] as the posterior becomes the prior and therefore reduce the divergence between the observed and predicted body state.

We argue that Sarah will not be able to infer other causes than pain for the incoming sensation. This might be due to individual goals and preferences in persons with chronic pain. Sarah might aim to regulate the “feared” interoceptive sensations rather than to accurately perceive the sensation (Farb et al., 2015) because she values a pain-free state more than people without chronic pain would and thereby the pain treatment that allows her to reach this state. Persons with chronic pain may also have a preference to overpredict so that they have fewer undertreated pain episodes. If Sarah pursues the goal to regulate interoceptive sensations, she will engage in active inference by down-weighting discrepant sensory information and generating confirmatory sensory input in favor of restoring a previously expected state (i.e., pain). Sarah’s brain will therefore more likely initiate visceromotor actions that actively generate the expected sensation (active inference, e.g., by rubbing or tensioning her stomach resulting in stronger sensations). This will then lead to Sarah actually perceiving pain as a result of the perceptual inference based on the new interoceptive sensations. Sarah might then engage in regulatory activities such as taking pain medication. Alternatively, Sarah might engage in worrying about her expected pain as a method to change her focus of attention (attentional shift). This worrying might serve three functions (Eccleston and Crombez, 2007): it might (a) activate alternative brain areas thereby decreasing the focus on the interoceptive prediction (Paulus and Stein, 2010; Seth, 2013; Barrett and Simmons, 2015), (b) serve to keep the physiological arousal under control associated with the increased anxiety as suggested by the avoidance model of worry (Borkovec et al., 2004), and (c) maintain vigilance to the expected pain and engagement to finding a solution (Eccleston and Crombez, 2007).

There are two core assumptions that we put forward: First, individuals with chronic pain will display a generally heightened pain prediction. Second and related to the first, they will generate an increased posterior probability of pain via active inference when harmless bodily sensations occur.

A large number of studies have addressed the question on how beliefs (such as anticipation of pain) exerts impact on what we perceive or see (for a critical review see Firestone and Scholl, 2015). Brown et al. (2014) recently showed augmented anticipation-induced potentials in patients with fibromyalgia to laser heat stimulation compared to patients with osteoarthritis and pain-free individuals, suggesting heightened pain predictions in situations where pain is anticipated. Heathcote et al. (2016) found that adolescents who catastrophized about pain were more likely to endorse negative interpretations of ambiguous situations, which we interpret as a consequence of their heightened pain prior expectations. Whether individuals with chronic pain display a continuous heightened pain prediction across various situations warrants further investigation. The assumption of a heightened pain prediction has been recently discussed from a fear learning perspective (Zaman et al., 2015). Zaman et al. (2015) provides a review of experimental and clinical studies providing evidence for a transition of non-painful to painful sensations after aversive conditioning (e.g., Wiech et al., 2010). Fear learning is assumed to account for this transition. Specifically, bodily sensations become through repeated associations with painful events predictive of pain and aversive themselves. This will foster predictions of pain. We suggest that these heightened pain predictions (Seth, 2013; Barrett and Simmons, 2015) will bias the perceptual process toward pain, resulting in an increased posterior probability of pain in the near future and thereby reinforcing the learned CS-US associations.

The reasoning behind the second assumption, i.e., that individuals with chronic pain will generate an increased posterior probability of pain via active inference when harmless bodily sensations occur, is that we sample the world to ensure our predictions become a self-fulfilling prophecy and surprises are avoided (Friston, 2009). Evidence for this second assumption is still scarce. Regarding active inference, Buchel et al. (2014) recently put forward the idea that in the context of placebo hypoalgesia, the ascending and descending pain system resembles a recurrent system that allows for the implementation of predictive coding. Specifically, they suggest that the brain is not passively waiting for nociceptive stimuli to impinge on it but is actively making inferences based on prior experience and expectations. The authors provide a review of findings in the context of acute pain. Tabor et al. (2015) provided evidence for the impact of predictions of the brain on perception of painful stimuli. Specifically, they could show that when people anticipate pain they underestimate the distance of the threat (painful) stimulus compared to a relief stimulus suggesting that pain-evoking stimuli are perceived as closer to the body.

## Research Agenda

The general hypothesis that individuals with chronic pain show heightened pain perception when confronted with harmless sensory input because of predictive coding process is new and will need to be elaborated and extended in future studies. There are several questions that warrant addressing: First, the hypothesis that individuals with chronic pain generate heightened pain predictions needs to be tested. Therefore, individuals with chronic pain should be investigated in various situations, e.g., during stressful situations or during situations that evoke sensations proximal to the main pain region. One way to study the assumption is to assess interoceptive accuracy across different levels of arousal, and across stressful situations. This has been successfully done in individuals with anorexia nervosa (Khalsa et al., 2015). They found that during meal anticipation individuals with anorexia nervosa experienced abnormal intense interoceptive sensations, although a low arousal level was induced indicating that prediction signals are abnormal at low arousal levels, especially during meal anticipation. Another way would be to develop a vignette-based task similar to Heathcote et al. (2016) to measure not only the posterior interpretation of ambiguous situations but also prior pain predictions.

Second, the exact mechanisms and conditions that lead from a heightened pain prediction to a heightened pain perception warrant further investigation. In the current paper, we restricted ourselves to a computational analysis of the process. The next step is to implement the model on an algorithmic level and to investigate how, assuming that perceptual inference works like Bayesian updating, this process is influenced by the patients’ likelihoods, i.e., their beliefs about the causal relationship between certain harmless bodily sensation and pain [*p(sensations|pain)*] as well as their beliefs about alternative causes for the sensations [p*(sensations|other causes)*]. This could be studied by explicitly querying the patient’s general causal beliefs of pain and interoceptive sensations and comparing them to beliefs of a healthy control group, or again by extending the approach of Heathcote et al. (2016).

Third, the next step would then be the use of computational simulations of the above-mentioned predictive coding strategies to explain the observed effects in individuals with chronic pain. Such models might also prove useful in predicting the expectable effect sizes of treatments which could target either the prior or the likelihood of pain.

In keeping with the general approach of computational psychiatry (Fletcher and Frith, 2009; Pellicano and Burr, 2012; Adams et al., 2013; Schwartenbeck and Friston, 2016), we thus suggest to derive quantitative models, infer their priors from human data in experimental paradigms and propose treatments from our normative Bayesian theory of interoception, that can then be tested experimentally.

Fourth, do these heightened pain predictions result in a chronic physical burden such as a chronic metabolic imbalance which is cused by constantly predicting the need for more metabolic energy to respond to the predicted pain? This imbalance might downregulate the HPA-axis resulting in chronic hypercortisolemia (Barrett and Simmons, 2015), indicative of a permanently altered stress response. Dysregulation of the HPA-axis have been found in individuals with chronic pain (Fukudo, 2013; Shahidi et al., 2015) but have not yet been investigated in the context of altered interoceptive predictions.

Finally, for an implementational level of active inference, one would have to appeal to neurobiologically plausible process theories for active inference (e.g., Friston, 2012; Barrett and Simmons, 2015), which is, however, beyond the scope of the current review.

## Conclusion

In chronic pain research, interest is growing into interoceptive processes, particularly into anticipatory anxiety of pain elicited by previously neutral interoceptive sensations. Here, we argued from a Bayesian perspective and formulate an application of recent neurocognitive and neuropsychological models to account for these maladaptive interoceptive processes in individuals with chronic pain.

In our view, the application of these theoretical models will broaden the present research and foster research into modeling the aberrant interoceptive predictions in individuals with chronic pain according to the Bayes theorem, into investigating the cognitive, emotional and behavioral consequences of the heightened pain predictions, and into the underlying mechanisms. In the long run, this research may foster testing the efficacy of interventions to modify the heightened pain perceptions of the mind. To achieve this goal, interventions to decrease the heightened pain prediction such as exposures to maximize the mismatch between expectancies and outcome as suggested by the expectancy violation model (Craske et al., 2011; Rief et al., 2015), could be combined with interventions that enable the individuals to change their causal attributions (of pain) for interoceptive sensations and reduce their active inference of pain (Jensen et al., 2014; Farb et al., 2015).

## Author Contributions

TH was responsible for the conception, drafting, and revising of the Perspective. DE and AT critically evaluated the adaptation of the interoceptive coding model to chronic pain research and revised the Perspective.

## Conflict of Interest Statement

The authors declare that the research was conducted in the absence of any commercial or financial relationships that could be construed as a potential conflict of interest.

## Footnotes

1 This is a generic case example, similar to previously reported ones.
